# Systemic lupus erythematosus‐related acute pancreatitis: An exceptional form with severe exocrine and endocrine pancreatitic failure in a Tunisian child

**DOI:** 10.1002/ccr3.5423

**Published:** 2022-02-11

**Authors:** Maryem Ferjani, Mounira El Euch, Yousra Hammi, Taha Sayari, Ouns Naija, Fethi Ben Hamida, Sami Turki, Tahar Gargah

**Affiliations:** ^1^ Pediatrics department Tunis Tunisia; ^2^ University of Tunis El Manar Tunis Tunisia; ^3^ Internal medicine department « A » Tunis Tunisia; ^4^ Research Laboratory of Kidney Diseases (LR00SP01) Charles Nicolle hospital of Tunis Tunis Tunisia

**Keywords:** child, lupus pancreatitis, pancreatitis, systemic lupus erythematosus

## Abstract

Acute pancreatitis may be the first manifestation in systemic lupus erythematosus or occur during evolution. It is a rare complication, which is often associated with other visceral manifestations. Outcome is usually favorable but can be serious. We report a case of a 17‐year‐old girl with a past history of systemic lupus erythematosus who developed acute pancreatitis revealed by abdominal pain. Elevated serum amylase and lipase levels and pancreatic enlargement on tomography confirmed the diagnosis. Although high‐dose corticosteroid was prescribed, the patient died from a refractory diabetic ketoacidosis.

## Graphical abstract

SLE‐related AP is a rare and severe condition. Diagnosis is based on amylasemia and abdominal scanner. Each practitioner has to focus on endocrine pancreatic failure which is severe and life‐threatening.

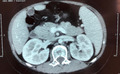

## 1 BACKGROUND

Acute pancreatitis (AP) is an acute inflammatory condition of the pancreas presenting among the gastroenterological emergencies of the abdomen.^1^ While alcohol and gallstones represent the frequent etiologies, involvement of the pancreas in systemic lupus erythematosus (SLE) is rare.^2^ Even in lupus, pancreatitis could result from different mechanisms including drug injuries or autoimmunity.^3^ Although diagnosis seems to be easy based on abdominal pain and elevated serum pancreatic markers, its therapeutic management and prognosis are variable.^4^ Unfortunately, pancreatic involvement is one of the life‐threatening clinical situations, and frequently it is a part of multiple organ damage like macrophage‐activated syndrome.^5^ We report an original case of a Tunisian child presenting with SLE complicated by pancreatitic global failure, macrophage‐activated syndrome, and refractory ketoacidosis.

## 2 OBSERVATION

**FIGURE 1:**
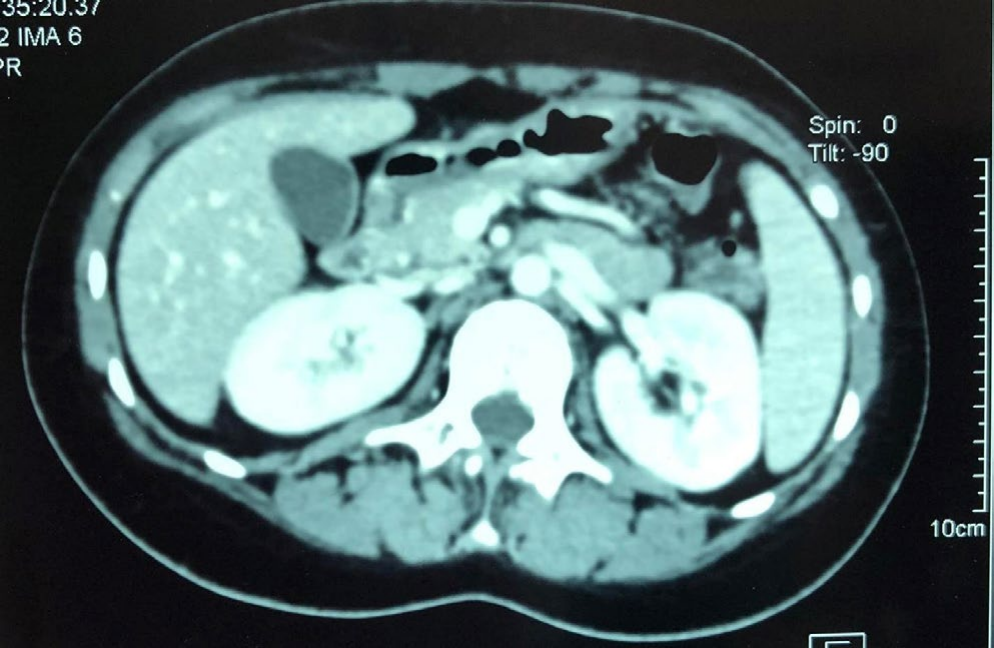
Abdominal tomography at first admission showing pancreatitis stage A

**FIGURE 2:**
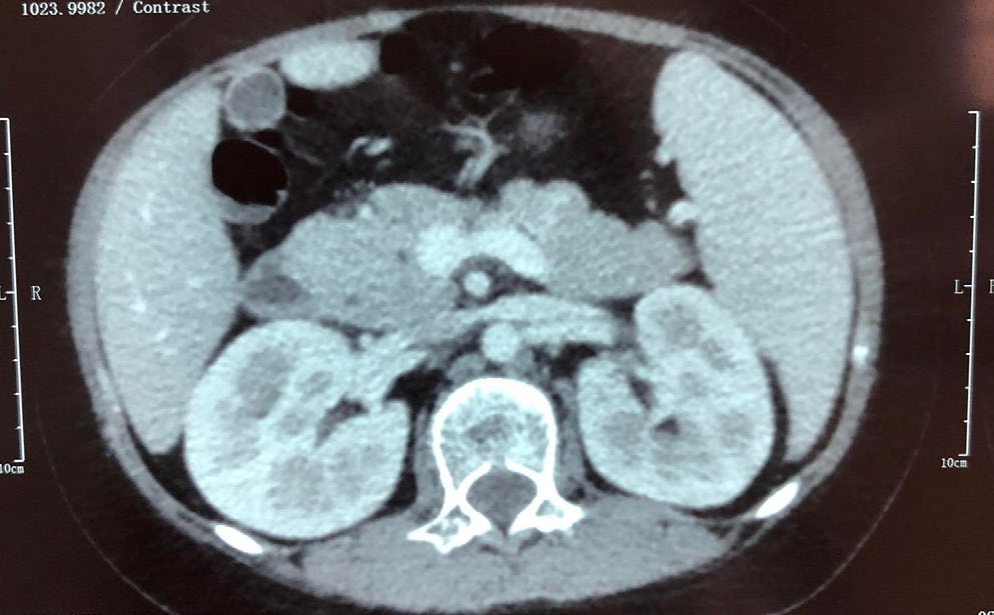
Abdominal tomography few days later showing aggravated pancreatitis to stage B

A 13‐year‐old girl followed for a SLE diagnosed with skin involvement made by malar rash, hemolytic anemia, leukopenia, lymphopenia, and elevated antinuclear autoantibodies rate at 1:800. She was treated first by photoprotection, antimalarial treatment, and corticosteroids with a favorable initial outcome. Four years later, she was admitted for acute abdominal pain, vomiting, and fever. On physical examination, the abdomen was painful without joint swelling or tenderness on extremities. Laboratory investigations were as following: hemoglobin 116 g/L, white cell count 1.75 × 10^9^/L (neutrophils 60%, lymphocytes 30%, and monocytes 5%), platelet count 150 × 10^9^/L, and C‐reactive protein less than 10 mg/L. Liver function analyses showed: alanine aminotransferase 31 U/L, aspartate aminotransferase 34 U/L, total bilirubin 2.4 µmol/L, triglyceride 2.54 mmol/L, alkaline phosphatase 119 U/L, amylase 87 U/L, and lipase 246 U/L. Immunological results revealed: antinuclear antibody 1:690, anti‐dsDNA antibody positive, anti‐Sm antibody negative, anti‐SS‐A antibody, and anti‐SS‐B antibody negative. Concentrations of complement components were very low: CH50: 3 U/L, C3: 0.2 g/L, and C4: 0.08 g/L. Abdominal tomography revealed acute pancreatitis stage A of Balthazar classification (Figure 1). SLE‐related AP diagnosis was retained regarding the elevated rates of auto antibodies and after excluding other frequent etiologies of pancreatitis. She was treated by gastroprotectors and high rates of corticosteroids at 1 mg/kg /day. Despite abdominal pain started vanishing and pancreatic level serum tests decreasing, she developed a severe hemolytic autoimmune anemia during her hospitalization, elevated both triglyceridemia and ferritin levels, with active hemophagocytois at bone marrow studies. Macrophage‐activated syndrome diagnosis was retained, and intravenous immunoglobulins were administrated. Unfortunately, she presented acute dyspnea and abdominal pain with increasing lipase test at 989 UI/L and aggravated pancreatitis to stage B at chest tomography of contrôle (Figure 2). Her dextro test showed 5.3 g/l of glycemia and high glucosuria with important acetonuria. Blood gazes revealed a marked metabolic acidosis. Although adequate electrolytic and glycemic correction protocol was immediately started, she developed quickly a refractory coma and died two days later.

## 3 DISCUSSION

AP is a very rare manifestation of SLE, especially in children. In adults, its incidence has been estimated between 0.4 and 1.1 cases per 1000 lupus per year.^6^ In Tunisia, the incidence has not been studied in children. A multicenter study of 232 children cohort including 14 centres in the UK followed over a period of four years showed that only 0.5% of them had pancreatic involvement.^7^

The pathogenesis of AP is multifactorial, and several etiologies are often incriminated like ischemic mechanism due to vasculitis or thrombosis related to an associated antiphospholipid antibody syndrome, autoimmune mechanism, or drug injury induced by corticosteroids and azathioprine or an intercurrent infectious disease.^8^ In our patient, AP was related to the SLE regarding immunological pertubances with concomitant multiorgan manifestations and after excluding frequent etiologies. In literature, acute pancreatitis is often consequent of alcohol or gallstones in the adult and possibility of pancreatic abnormality in childhood.^9^

Clinical manifestations of our patient are very similar to literature based on abdominal pain, reported in most of the patients. In some reports, AP could be asymptomatic based on biological or radiological findings.^10^

At biology, elevated, elevated pancreatic serum tests associated or not to cholestasis or cytolysis is the most common situation with exocrine pancreatic failure or hypertriglyceridemia.^11^ The particularity of our case was the endocrine pancreatic failure associated which has been reported in only one report with a severe diabetes secondary to the AP.^12^

Radiological tests showed initially normal pancreatic gland seizure which increased quickly. These findings have been reported by many similar cases which described the progressive extension of inflammatory process in the pancreatic gland.^13^

The occurring of macrophage‐activated syndrome worsened the prognosis of AP which was also reported in several publications especially in childhood and must be focused on by checking ferritin and triglyceridemia.^14^

Unfortunately, we do not have an exact therapeutic consensus in AP given its rarity especially in children. Corticosteroid therapy should be maintained and in high doses; unless if it is formally incriminated in the occurrence of AP. It is indicated especially with vasculitis and to improve the clinical and biological abnormalities.^3^ Other studies combined immunosuppressive drugs like cyclophosphamide or azathioprine, plasmapheresis sessions, and intravenous immunoglobulin pulses with favorable outcome.^15^ Although our patient received human immunoglobulin at 2 g/kg in addition to corticosteroid therapy, we noticed a worsening of the exocrine (from stage A to B of Balthazar classification) and endocrine pancreatitis revealed by diabetic ketoacidosis.

The prognosis of AP can be fatal due to necrosis and hemorrhagic forms or specific complications^16^ such as ketoacidosis developed by our patient. In literature, even if acute complication does not occur, patients develop severe uncontrolled diabetes.^12^ To avoid such severe complications, some authors insist on the importance of the systematic screening of AP to make early diagnosis and treatment.^16^, ^17^

## 4 CONCLUSION

Our original case illustrated an exceptional SLE‐related AP complication which is the endocrine deficiency. AP should be usually evocated in SLE regarding abdominal pain, elevated lipase serum, and radiological specific findings after excluding other etiologies. The occurring of macrophage‐activated syndrome worsened the prognosis of SLE related to AP which could be favorable with combination of steroids, immunosuppressive drugs, or immunoglobulin. The focus of glycemic blood and urinary tests must be enrolled in the follow up of these patients to screen about this severe complication and avoid mortality.

## CONFLICT OF INTEREST

Authors do not declare any conflict of interests.

## AUTHORS CONTRIBUTION

MF: conceived the study and wrote the paper. MEE: helped writing the paper. YH: performed experiments. STa: analyzed data. NO and STu: contributed to the management of the patient. FBH: financed the project. TG: supervised and aprobated the project.

## ETHICAL APPROVAL

Mother's consent has been obtained before submission.

## CONSENT

Written informed consent was obtained from the patient's mother to publish this report in accordance with the journal's patient consent policy.

## Data Availability

Clinical, biological and radiological data are available on medical file of the patient in pediatrics department.
